# Wavelet denoising of fiber optic monitoring signals in permafrost regions

**DOI:** 10.1038/s41598-024-59941-4

**Published:** 2024-04-20

**Authors:** Bowen Ni, Fei Song, Liguo Zhao, Zhipeng Fu, Yongyi Huang

**Affiliations:** 1 School of Highway Engineering, Institute of Geotechnical Engineering, Chang’an University, Xi’an, People’s Republic of China; 2 CCCC First Highway Consultants Co., Ltd, Xi’an, People’s Republic of China

**Keywords:** Geotechnical engineering, Optical fiber monitoring, Wavelet denoising, Engineering, Mathematics and computing

## Abstract

To address the noise issue in fiber optic monitoring signals in frozen soil areas, this study employs wavelet denoising techniques to process the fiber optic signals. Since existing parameter choices for wavelets are typically based on conventional environments, selecting suitable parameters for frozen soil regions becomes crucial. In this work, an index library is constructed based on commonly used wavelet basis functions in civil engineering. An optimal wavelet basis function is objectively selected through specific criteria. Considering the characteristic of small root mean square error in fiber optic signals in frozen soil areas, a multi-index fusion approach is applied to determine the optimal decomposition level. Field observations validate that denoised signals, with parameters set appropriately, can more accurately identify locations where settlement occurs.

## Introduction

In the permafrost regions of the Qinghai-Tibet Plateau, the extreme climatic conditions characterized by high altitude and latitude pose significant challenges to engineering development and maintenance during the construction of the Qinghai-Tibet Highway. Traditional monitoring methods incur prohibitively high operational costs in these frozen soil areas. In this context, fiber optic monitoring technology proves particularly advantageous for extensive and remote measurement point distribution on highways^1^. With its remarkable adaptability, fiber optic monitoring technology offers an advanced solution for monitoring in permafrost regions^2^.

However, adverse weather conditions in these areas may introduce interference and noise into fiber optic monitoring data, significantly affecting monitoring accuracy and potentially compromising judgment on monitoring results. Therefore, research on fiber optic monitoring noise reduction techniques is crucial, as it enhances the quality of monitoring data, ensuring the safe operation of engineering structures in permafrost regions.

Wavelet analysis, emerging in the mid-1980s as a time-domain localization analysis method, is renowned for its multi-resolution analysis capabilities and outstanding noise reduction performance^3^. Research on wavelet denoising techniques has a long history of continuous improvement, particularly in engineering applications. Wang et al.^4^ demonstrated the effectiveness of wavelet denoising algorithms in reducing noise in fiber optic monitoring .

Ahmed Silik et al.^5^ considered various evaluation factors to select the optimal wavelet, yet the subjective process of choosing suitable wavelet functions still requires experiential support. Moreover, no validation results for practical structural applications were provided. Byungil Kim et al.^6^ explored the application of wavelet transforms in civil engineering and provided guidance on selecting appropriate wavelet types.

Srivastava et al.^7^ proposed a method related to the selection of thresholds and decomposition levels. Jiang et al. used sparse indicators to select appropriate wavelet parameters.

It is worth noting that the majority of existing research findings and experimental data have been obtained under conventional environmental conditions. When dealing with frozen soil areas characterized by high altitude and cold climates, unique environmental conditions may introduce unknown impacts on signals. The applicability of wavelet denoising techniques in permafrost regions remains unverified. Additionally, parameter optimization for existing wavelet denoising techniques has been concluded under conventional environmental conditions, with the selection of most wavelet parameters determined based on evaluation indicators such as SNR and RMSE^8–10^. However, the RMSE of fiber optic monitoring data in permafrost regions is extremely low, rendering RMSE as an ineffective evaluation metric. Consequently, there is limited research addressing this specific scenario.

To validate the effectiveness of wavelet denoising in permafrost regions, a meticulous reassessment of parameters such as the choice of appropriate wavelet basis functions, decomposition levels, and thresholds is necessary. This is crucial for further elucidating the application scope of wavelet denoising techniques and enhancing the precision of fiber optic monitoring in permafrost regions.

## Methods

The process of wavelet denoising aims to eliminate noise that is mixed into high-frequency signals, allowing the recovery of low-frequency or true signals. During the denoising process, the choice of wavelet functions, threshold selection criteria, thresholding methods, and decomposition levels all significantly affect the denoising performance^11^. The effectiveness of wavelet denoising relies on the low entropy, multi-resolution characteristics, decorrelation properties, and flexibility in wavelet basis function selection.

Therefore, the selection of suitable wavelet basis functions and reasonable decomposition levels is of paramount importance. Most existing research has concentrated on the selection of wavelet basis functions for monitoring results in normal temperature environments. However, for the frigid and high-altitude frozen soil regions, it is imperative to reevaluate wavelet denoising methods and conduct in-depth analysis.

The process of wavelet denoising primarily comprises three main steps: signal decomposition, threshold quantization, and wavelet reconstruction.

In the first step, an appropriate wavelet basis function and decomposition level are selected to perform multi-scale wavelet decomposition on the signal. This step results in the derivation of wavelet coefficients at each scale.

The second step involves selecting suitable threshold functions and thresholds for threshold quantization of the high-frequency coefficients at each decomposition scale.

In the final step, wavelet reconstruction is performed using the lowest-level low-frequency coefficients and the high-frequency coefficients from all layers, yielding the denoised signal.

Key factors affecting the denoising effectiveness encompass the choice of decomposition scale, wavelet basis function, threshold criteria, and threshold quantization function. Therefore, when determining the filtering approach for frigid regions, it is essential to carefully consider the aforementioned factors.

### Selection of wavelet basis functions

In wavelet analysis, there are many types of mother wavelet which can be used for wavelet analysis. Different mother wavelet used to analyze the same signal will produce different results. Generally, mother wavelets are characterized by properties such as orthogonality, compact support, symmetry and vanishing moment. Based on previous study, properties of mother wavelet are considered in selecting a mother wavelet. However, more than one mother wavelet with the same properties often exists. To overcome this, the similarity between signal and mother wavelet are considered in selecting a mother wavelet^12^.

Among the commonly used wavelet basis functions are the Haar wavelet, Daubechies wavelet series, Biorthogonal wavelet series, Coiflet wavelet series, Symlets wavelet series, Morlet wavelet, Mexican Hat wavelet, and others.

The wavelet basis is characterized by five crucial parameters^13–15^: orthogonality, symmetry, regularity, vanishing moment, and compact support. Orthogonality denotes the perfection of the wavelet basis, providing insights into data redundancy and rigorously governing its orthogonal attributes. This characteristic fosters precise reconstruction of wavelet decomposition coefficients. Symmetric wavelet bases, characterized by linear phase properties, yield filter banks that exhibit insensitivity to symmetric quantization errors near edges, mitigating phase distortion during signal decomposition and reconstruction.

Regularity articulates the differentiability of the wavelet basis, serving as an indicator of the smoothness of the wavelet function. It plays a vital role in effectively identifying singular points in signal wavelet transforms. Higher regularity in most orthogonal wavelet bases corresponds to an increased number of vanishing moments.

The vanishing moment quantifies the convergence rate of wavelets when approximating a smooth function, providing a measure of energy concentration post-wavelet transformation.The magnitude of the vanishing moment influences the convergence rate, signifying the degree of energy concentration following the wavelet transformation.

The support width reflects the localization capability of the wavelet basis. Smaller support widths enhance the localization ability of the wavelet basis. In the calculation of wavelet transforms, lower complexity and larger support contribute to improved regularity^16^.

Table 1 shows the parameters corresponding to commonly used wavelet bases.

**Table 1:** Comparison of common wavelet basis parameter characteristics.

Wavelet basis functions	Orthogonality	Symmetry	Vanishing moment	Support length
Haar	Yes	Symmetrical	1	1
Daubechies (db N)	Yes	Approximately symmetrical	N	2N − 1
BiorSplines	No	Asymmetrical	Nr − 1	2N + 1
Coiflets (coif N)	Yes	Approximately symmetrical	2N	6N − 1
Symlets (sym N)	Yes	Approximately symmetrical	2N	2N − 1

It is important to strike a balance, as excessively long support lengths may hinder the local characteristics of wavelet analysis and algorithm implementation. In this regard, the Daubechies wavelet series and Symlets wavelet series are noteworthy choices.

Simultaneously, relevant studies indicate that using Symlets and Daubechies as wavelet basis functions for wavelet denoising is more effective than other wavelet basis functions, especially when the number of decomposition layers is three, yielding optimal wavelet denoising performance^5^. Kim conducted a statistical analysis of the mother wavelets selected for wavelet denoising in the field of civil engineering. There are six specific applications in civil engineering: denoising, discontinuity detection, feature extraction, frequency recognition, system modeling, and data compression. This analysis aims to assist civil engineers in choosing the appropriate wavelet transform type for specific aspects of civil engineering. The results indicate that the db wavelet is the most frequently used^6^.

To further select the most suitable wavelet basis functions for the signals in high-altitude and cold regions, it is necessary to introduce a quantitative method for evaluating the Symlets (sym) and Daubechies (db) wavelet basis functions.

However, in permafrost regions, commonly used metrics for evaluating the applicability of wavelets, such as MSE and RMSE, exhibit extremely low values after signal denoising under Daubechies (db) and Symlets (sym) wavelet decompositions. This poses challenges in conducting comprehensive assessments to identify the optimal indicators. In response to this issue, Cunha et al. proposed a method for selecting the optimal wavelet based on maximizing the signal-to-noise ratio (SNR). The efficacy of this method was compared to the Energy-Based Wavelet Selection (EBWS) method and the Correlation-Based Wavelet Selection (CBWS) method. Results demonstrated that the proposed algorithm exhibited superior performance^17^.This study compares the Symlets (sym) and Daubechies (db) wavelet basis functions, which are commonly used in the field of civil engineering, using the aforementioned method.

Constructing a comprehensive library of all Symlets (sym) and Daubechies (db) wavelet functions, with other parameters held constant. Systematically calculating the approximation coefficients (ai) and detail coefficients (di) for the actual measured signal through the entire wavelet library. Compute the Signal-to-Noise Ratio (SNR) in decibels (dB) using Eq. (1) for each pair of coefficients and select the mother wavelet function that yields the maximum SNR value.

1 $${\text{SNR = 1 0 * log (max (signal) / max (noise) ) }}$$

Where the numerator is the maximum value of the detail or approx imation coefficients which present the larger peak magnitude, seen as coefficients of the signal of interest, and the denominator is the maximum value of the coefficients of lower peak magnitude, interpreted as noise coefficients.

The wavelet library utilized in this study comprises Daubechies wavelets of orders 2–25 and Symlet wavelets of orders 2–15. Employing the previously described calculation approach, SNR is systematically computed for each mother wavelet across various decomposition levels (3–8), aiming to identify the one that maximizes the SNR. The results are shown in Table 2.

**Table 2:** Under different decomposition levels, the optimal wavelet basis functions.

Decomposition level	3	4	5
Wavelet	db22	sym11	db8
SNR	17.8793	16.3779	14.8747
Decomposition level	6	7	8
Wavelet	db11	db25	db21
SNR	13.3713	11.8716	10.3708

**Figure 1:**
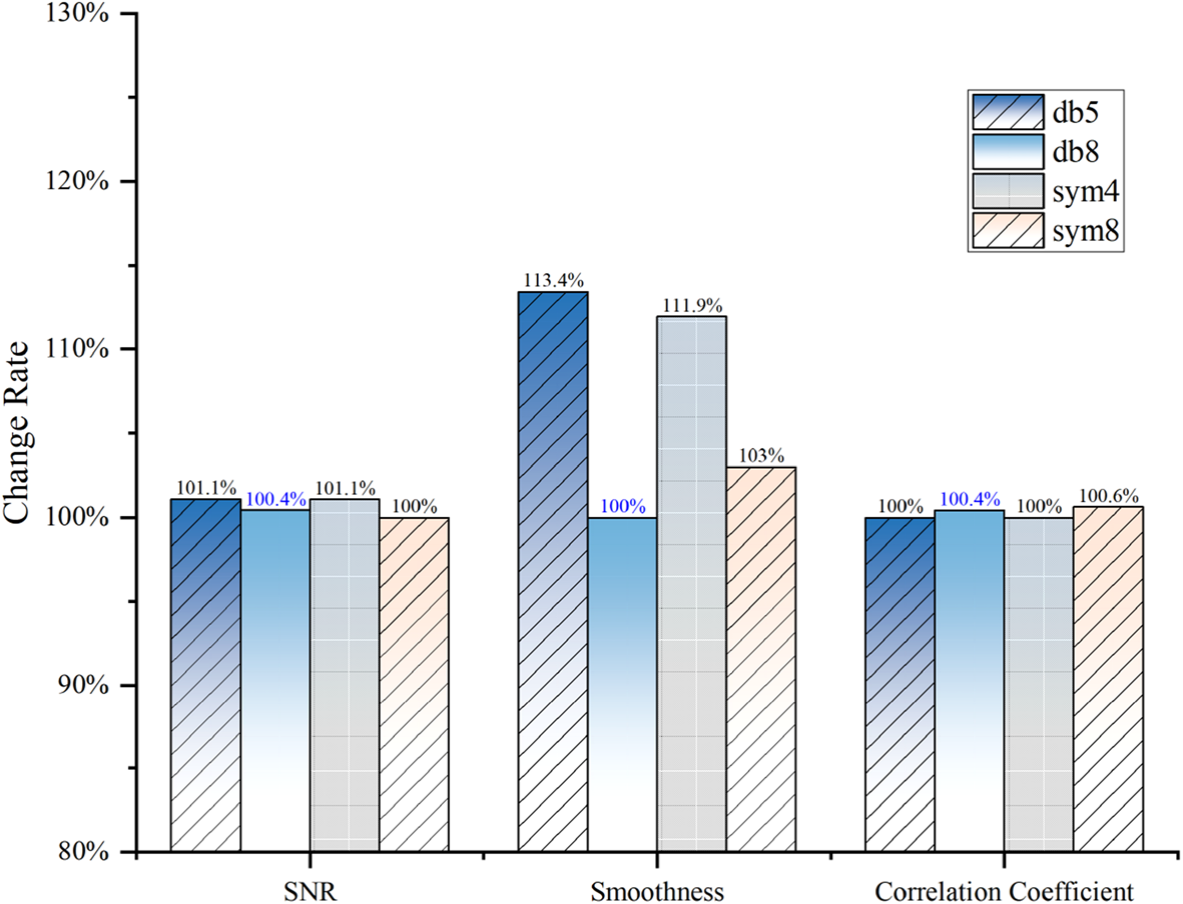
The comparison of denoising metrics between the optimal wavelet basis function and other commonly used wavelet basis functions.

To validate the effectiveness of this method, we selected the optimal wavelet db8 at the fifth level and compared it with several other commonly used wavelet basis functions under fixed threshold denoising for the same set of frozen soil fiber optic data. The results of the relevant evaluation parameters are depicted in Fig. 1. It can be observed that db8 outperforms the other parameters significantly in terms of the smoothness indicator.

### Selection of wavelet decomposition levels

The number of decomposition levels in wavelet processing directly influences the denoising effectiveness. If the number of levels is too low, a significant amount of noise will remain in the signal. Conversely, if the number of levels is too high, some detailed information in the signal may be treated as noise, while also increasing computational complexity without a significant improvement in denoising effectiveness. Therefore, selecting an appropriate number of decomposition levels is crucial^18^.

For the selection of an appropriate number of decomposition levels, commonly used evaluation metrics in wavelet threshold denoising include Signal-to-Noise Ratio (SNR), Root Mean Square Error (RMSE), and smoothness (r). SNR, defined as the energy ratio between the original signal and the noise signal, indicates better denoising performance with larger SNR values. RMSE, representing the square root of the variance between the original signal and the denoised signal, reflects better denoising performance with smaller RMSE values. Smoothness, calculated as the ratio of squared differential values between the denoised signal and the original signal, characterizes the local variations in the denoised signal, with smaller values indicating better denoising effectiveness.

However, relying on a single criterion for evaluation has inherent limitations. For instance, in cases where the true values are unknown and environmental noise is predominantly low-frequency, the root mean square error (RMSE) after denoising may become very small or approach zero. When using signal-to-noise ratio (SNR) as a sole criterion for evaluation, it exhibits a decreasing trend with an increase in decomposition levels. Consequently, accurately determining the optimal denoising effectiveness becomes challenging.

Srivastava determined the required denoising decomposition levels by observing the correlation between detail and approximation components^7^.

This method is based on the assumption that signals tend to appear consistently in the same locations with larger amplitudes, while random noise appears inconsistently with smaller amplitudes. However, a drawback of this method is its reliance on subjective judgment, requiring a certain level of experience. Therefore, a more objective criterion would ensure the reliability of the results. In formulating a composite index, it is imperative to comprehensively consider signal characteristics.

This study utilized a set of field-measured fiber optic data from high-altitude and cold regions. Employing the same wavelet basis function and constant threshold, different decomposition levels were applied to denoise the fiber optic data. Subsequently, the SNR, RMSE, and smoothness were recorded at various levels. The results are shown in Table 3.

**Table 3:** Signal evaluation results at various decomposition levels.

Wavelet decomposition levels	SNR	RMSE	Smoothness
3	73.09	0	0.7
4	68.52	0	0.49
5	66.63	0.01	0.45
6	64.73	0.01	0.43
7	59.53	0.01	0.42
8	57.99	0.01	0.41

Given the phenomenon of Root Mean Square Error (RMSE) approaching zero after denoising in high-altitude and high-latitude regions, employing RMSE as an evaluation metric may not distinctly reveal the quality of the signal.

This study employs two metrics, smoothness and signal-to-noise ratio (SNR), for comprehensive evaluation. The rationale behind selecting these two metrics lies in SNR’s ability to reflect detailed changes in the signal, while smoothness focuses on the overall trend of the signal.

In order to determine suitable weight coefficients for Signal-to-Noise Ratio (SNR) and smoothness, this study employs an objective weighting method known as the Coefficient of Variation (CV). Unlike subjective weighting methods, which rely on personal judgment and experience, the CV method is grounded in mathematical theory and offers greater reliability.

The Coefficient of Variation is a commonly used statistical measure to assess the variability of data across different evaluation objects. It quantifies the degree of variation in observed values for each metric. By considering the variability of each metric, we can objectively assign appropriate weights.

In summary, through the application of the CV method, this study aims to find an optimal combination of denoising effectiveness by assigning reasonable weights to SNR and smoothness, thus enhancing the reliability of the evaluation process.

Due to the disparate ranges of signal-to-noise ratio (SNR) and smoothness, normalization is required to ensure both metrics fall within the [0–1] range.

The normalization method is as follows:

2 $$Pr=\frac{r-min(r)}{max(r)-min(r)}$$

3 $$Psnr=\frac{snr-min(snr)}{max(snr)-min(snr)}$$

The weighted average can be calculated using the coefficient of variation (CV), which is defined as the ratio of the standard deviation (σ) to the mean (μ), expressed as:

4 $$CV=\frac{\sigma }{\mu }$$

The formula for calculating the weights (W) is as

5 $${W}_{Pr}=\frac{{CV}_{Pr}}{{CV}_{Pr}+{CV}_{Psnr}}$$

To obtain a new composite indicator (OPT) by linearly weighting the two normalized data sets (P_SNR_ and P_R_) :

6 $${\text{OPT}}=W{\text{PSNR}}\times {{\text{P}}}_{{\text{SNR}}}+W{\text{Pr}}\times {\text{Pr}}$$

To mitigate potential biases resulting from the limitations of a single weighting method, the present study explores an alternative objective weighting approach: the entropy weighting method.

To calculate the entropy value for the “smoothness” indicator:

7 $${E}_{{\text{r}}}=-\sum \limits_{j=1}^{n}\frac{{p}_{rj}ln{p}_{rj}}{lnn}$$

To calculate the weight of indicator:

8 $${w}_{{\text{r}}}=\frac{1-{E}_{{\text{r}}}}{{\sum }_{i=1}^{n}(1-{E}_{{\text{r}}})}$$

To obtain C by linearly weighting the two normalized data sets (P_SNR_ and P_R_) :

9 $${\text{C}}= {w}_{{\text{r}}}\times {{\text{P}}}_{{\text{r}}}+{w}_{{\text{snr}}}\times {{\text{P}}}_{{\text{snr}}}$$

Without altering the mother wavelet, threshold, and filtering methodology, the computation of OPT values and C values across various decomposition levels can be performed. The Table 4 showcases the calculated OPT values and C values for different decomposition levels.

**Table 4:** OPT and C values.

Wavelet decomposition levels	OPT	C
3	1	1
4	0.51564	0.50755
5	0.18354	0.33529
6	0.11033	0.26454
7	0.06442	0.19362
8	0	0

**Figure 2:**
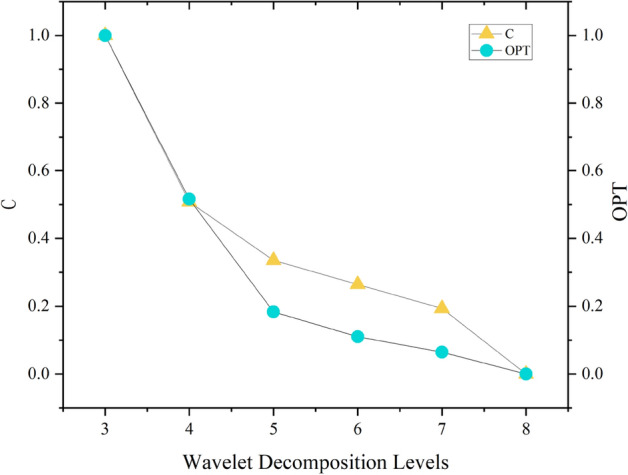
The trend of OPT and C over different decomposition levels.

According to relevant studies^19^, evaluation metrics for signal denoising demonstrate convergence. Significant decreases in the rate of change are observed upon reaching the optimal decomposition scale. Parameters such as OPT and C, which are composed of weighted combinations of multiple indicators, also exhibit convergence. Compared to the results obtained using entropy weighting, the convergence trend of OPT is more pronounced, suggesting that the singular value weighting method is more suitable for the weighting of the two indicators than the entropy weighting method. As shown in Fig. 1, the computed OPT demonstrates clear convergence. Prior to a decomposition level of 5, the OPT metric undergoes dramatic changes, whereas the rate of change gradually diminishes after the 5th level Fig. 2.

**Figure 3:**
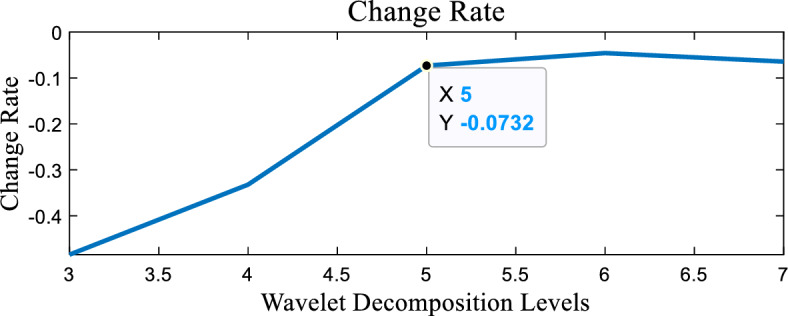
The change rate of OPT.

The change rate of OPT, as illustrated in Fig. 3, exhibits an inflection point at level 5, indicating that the 5th level is the optimal decomposition level. This conclusion aligns with the findings of Srivastava^7^.

### Selection of the threshold

To eliminate noise from the signal obtained through wavelet decomposition, it is necessary to filter out the detail components. These details include information signals with large coefficients and worthless noise signals with small coefficients. By setting a threshold, meaningless noise can be eliminated while retaining valuable signals. Donoho^20^ introduced four commonly used thresholding methods: sqtwolog, heursure, rigrsure, and minimaxi. The thresholds for sqtwolog (7), rigrsure (8), and minimaxi (9) can be calculated using the following formulas.

SQTWolog thresholding is suitable for wavelet thresholding in scenarios characterized by low levels of noise. In such contexts, where the disparity between signal and noise is relatively small, a more flexible thresholding approach is required to effectively attenuate noise while preserving significant signal features. SQTWolog thresholding exhibits a degree of adaptability, allowing for the dynamic adjustment of thresholds based on signal characteristics and noise levels.

10 $$th = \sigma \sqrt {\text{(2log(N))}}$$

The RigrSure method employs rigorous statistical principles and risk estimation to determine thresholds, thereby mitigating subjectivity and uncertainty inherent in threshold selection to a certain extent. This characteristic has led to widespread adoption of RigrSure thresholds in wavelet thresholding applications.

However, the performance of the RigrSure method may be sensitive to parameter selection, including the distribution characteristics of the signal and noise, as well as threshold adjustment parameters. Improper parameter selection could potentially compromise the accuracy and efficacy of threshold selection.

11 $$th = \sigma \sqrt {{\text{(W}}_{b} {)}}$$

The core concept of the Minimaxi method involves minimizing the average maximum deviation of the signal to determine thresholds. This approach effectively reduces the potential maximum error introduced during threshold processing, thereby preserving the local structure and key features of the signal. However, due to limited understanding of background noise, which may lead to excessive retention of signal detail.

12 $$th = \left \{ \begin{array}{*{20}ll} {\sigma \left( {0.3936 + 0.10829{\text{log}}_{2} N} \right)} & \quad {N > 32} \\ 0 & \quad {N < 32} \\ \end{array} \right \}$$

Where σ is the standard deviation of the noise signal, N is the length of the noise signal, and wb is the square of the b-th coefficient of the wavelet. The Heursure threshold selection rule is a combination of the Sqtwolog and Rigrsure methods^21^.

Due to the unknown nature of background noise, the original signal is filtered through four different thresholding methods.The similarity between the processed waveform and the waveform of the original signal can be used to determine a relatively appropriate thresholding method.To better preserve the local features of the signal’s edges, a hard thresholding filter was chosen. 

After employing various thresholding techniques for denoising, the evaluation metrics of the signal are presented in the following table. Although the other three thresholding methods exhibit slightly higher SNR values compared to SQTWolog, they notably demonstrate worse values in terms of smoothness and Correlation Coefficient parameters.The correlation coefficient refers to the similarity between the signal obtained after wavelet denoising and the original signal. It is generally believed that as the correlation coefficient approaches 1, the denoising effectiveness improves.

**Figure 4:**
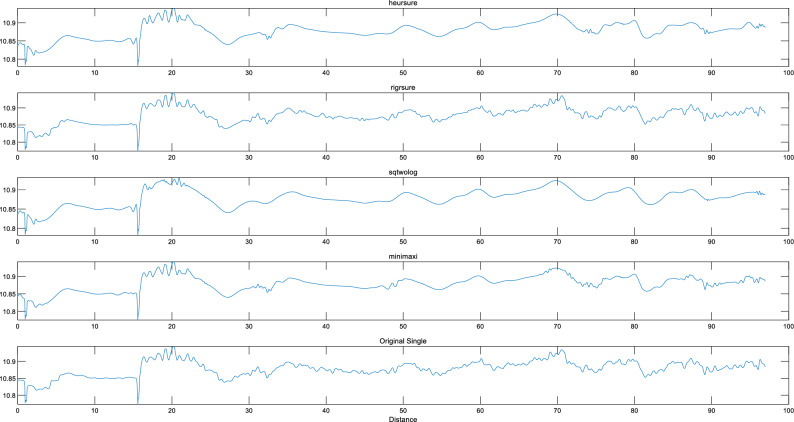
the original signal and the denoised signals.

As shown in the Fig. 4 and Table 5, for cases where the original signal exhibits small fluctuations, using a fixed threshold filtering method based on signal length is more suitable compared to the other three filtering methods.Considering that the median value of the monitoring data does not change extensively, the fixed threshold is appropriate^20^.

**Table 5:** The evaluation metrics following denoising with various thresholding strategies.

Thresholding methods	SNR	SNR change rate	Smoothness	Smoothness change rate	Correlation coefficient	Correlation coefficient change rate
SQTWolog	65.9	0	0.67	0	0.478	0
Rigrsure	76.44	16%	0.98	46%	0.466	− 2.5%
Minimaxi	68.63	4%	0.83	24%	0.471	− 1.5%
Heursure	67.73	3%	0.77	15%	0.473	− 1%

### Distributed fiber optic monitoring data for frozen soil roadbed deformation

#### Overview of engineering case study

The Qinghai Provincial Highway from Gonghe to Yushu traverses altitudes ranging from 3500 to 4700 m and spans the Yellow River and Yangtze River watersheds. Situated on the edge of the permafrost zone of the Qinghai-Tibet Plateau, the region represents a high-altitude, cold, and thermally unstable permafrost area in the mid- to low-latitude zone. Monitoring data of permafrost ground temperatures along the route indicate that the annual average ground temperatures of the permafrost range from − 0.1 to − 1.8 °C.

Based on the results of the survey, the permafrost sections along the route have a total length of 233.318 km. This includes segments with less ice and more ice permafrost covering 132.385 km, as well as sections with rich ice, saturated ice permafrost covering 94.525 km. Additionally, there are segments with soil containing ice layers covering 6.408 km. The remaining sections consist of either permafrost thaw zones or seasonal frozen soil areas.

This highway holds significance as it is the first highway in China that traverses through the permafrost region of the Qinghai-Tibet Plateau, serving as a major transportation route to the Yushu region.

**Figure 5:**
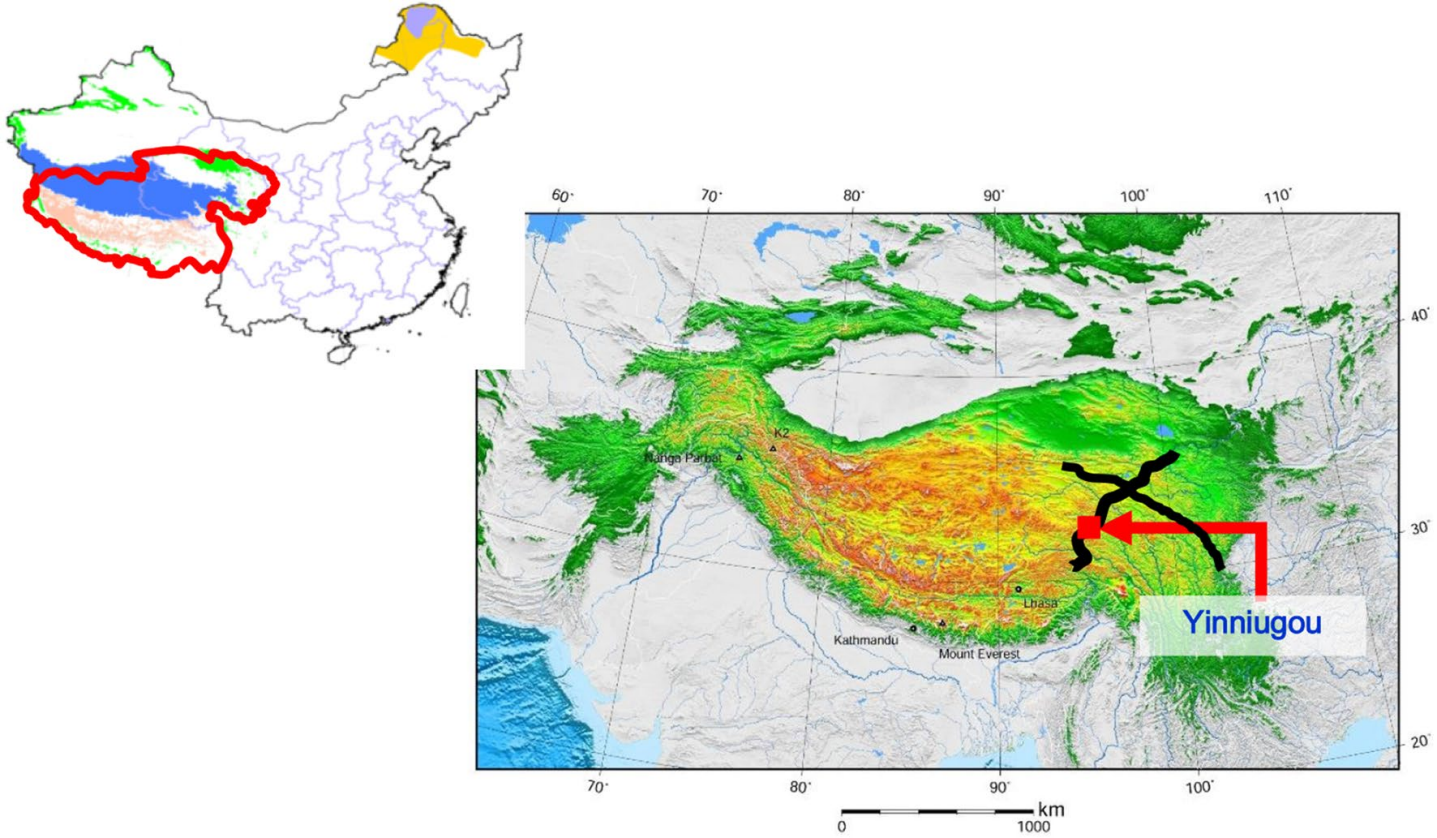
Map of monitoring site location.Map showing the location of NOAA surveys collected and U.S. Geological Survey (USGS) surveys collected along with sample and bottom photo locations.

This project selects the Yiniugou section of Gonghe-Yushu Highway in Qinghai Province (milepost: K569 + 540–K569 + 640, K569 + 640–K569+740, K570 + 300–K570 + 400) for application. The specific project location is shown in the Fig. 5.The Yiniugou section of the highway is designed according to the standards of separated roadbed, with a roadbed top width of 12.25 m, 2% cross slope for driving lane, curb, and hard shoulder, and 4% cross slope for shoulder. The average altitude of this section is greater than 4500 m, and it belongs to the alluvial plain region. The terrain is flat and open, and the surface soil is mostly marshy wetlands or meadow soil. The types of permafrost are little ice-bearing permafrost, much ice-bearing permafrost, rich ice-bearing permafrost, saturated ice-bearing permafrost, and soil-ice layer. Among them, the length of rich ice-bearing permafrost and above is 5.242 km, accounting for 65% of the section. The upper limit of natural permafrost is 2–3 m, and the annual average ground temperature of permafrost is − 0.27 to − 1.7 °C. The proportion of high ice content permafrost is large, and the permafrost types and ground temperature variations are diverse, with complex surface conditions and strong representativeness and typicality, which can carry out monitoring and demonstration of permafrost roadbed and pavement condition evaluation.

### Monitoring implementation plan

**Figure 6:**
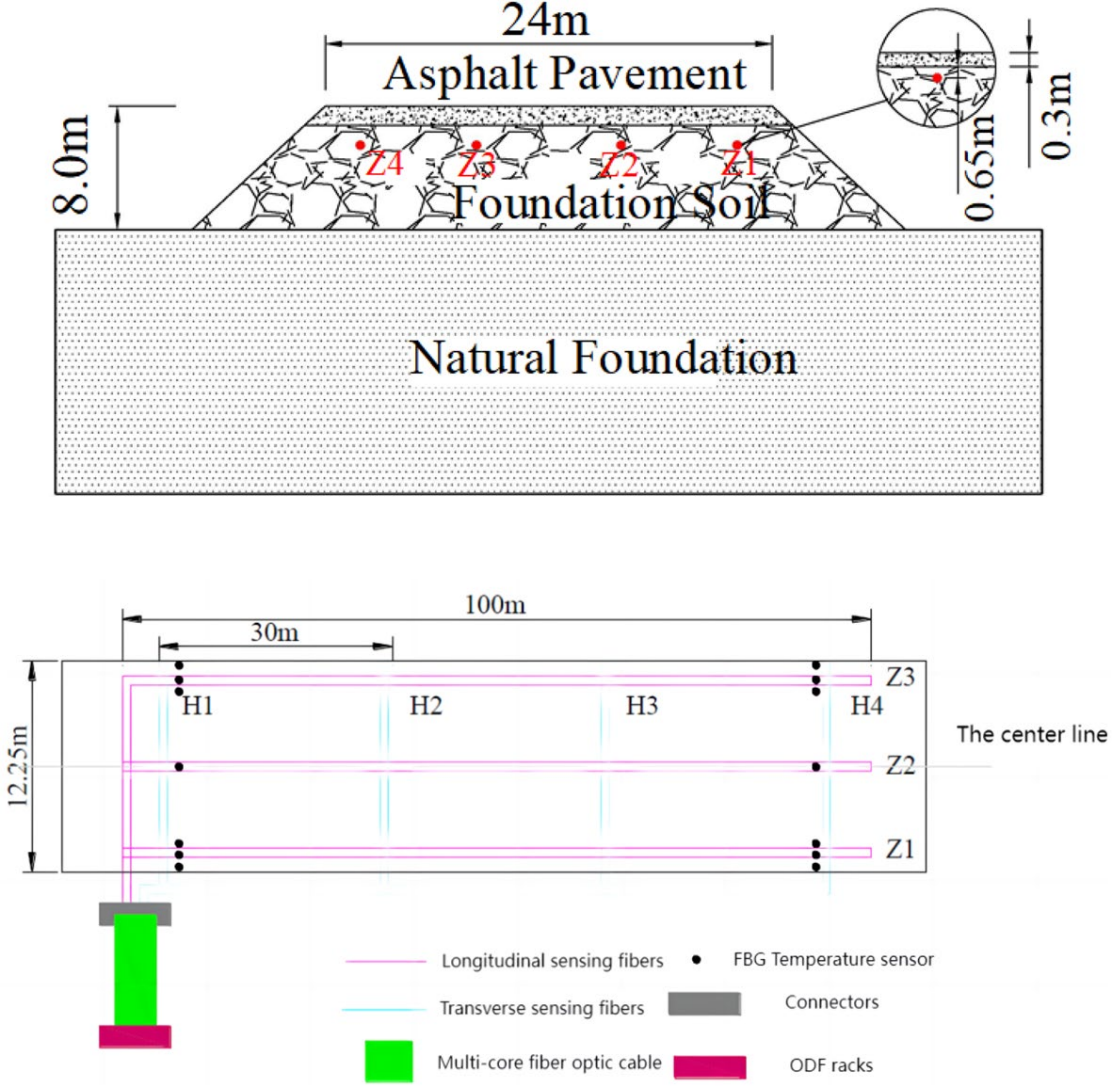
Fiber optic arrangement method.

The optical fiber sensors are buried as shown in Fig. 6. The selected optical cable is a metal-based helical strain sensing optical cable. This type of sensing optical fiber features a metal-based helical structure, with high-strength metal reinforcement, significantly enhancing the tensile strength of the sensing optical fiber. Additionally, the threaded structure on the sensor’s surface ensures good coupling with the surrounding soil, ensuring consistent deformation coordination.

### Illustrative example of proposed method

Based on the aforementioned analysis, the application of Daubechies (db) wavelet basis, a 5-level decomposition, and fixed threshold hard-threshold filtering were used to filter the original monitoring data.

**Figure 7:**
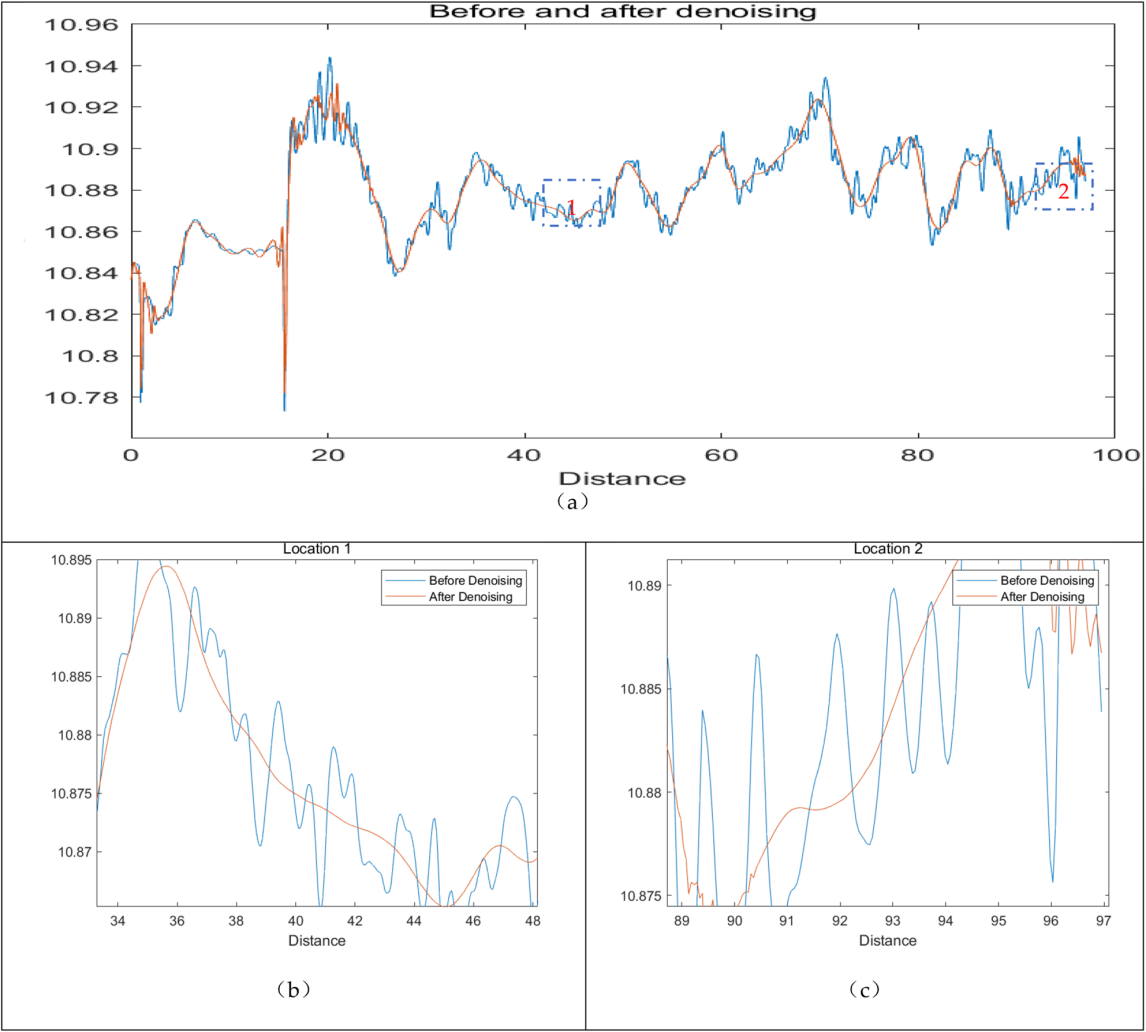
Before and after denoising: (**a**) A global version; (**b**) A larger version at location 1; (**c**) A larger version at location 2.

A comparison was made with the original data shown in Fig. 7. Compared to the original data, this approach effectively removes noise, resulting in smoother data while preserving the local variations patterns.

Before and after denoising, the signal’s mean squared error (MSE) remains unchanged, while the correlation coefficient of the signal approaches 1. The correlation coefficient(${r}_{xy}$) refers to the similarity between the wavelet denoised signal and the original signal.It is a commonly used metric for evaluating the quality of wavelet denoising.The calculation results are presented in Table 6.

13 $$r_{xy} = \frac{{\sum_{i = 1}^{N} \left( {x_{i} - \overline{x}} \right)\left( {y_{i} - \overline{y}} \right)}}{{\sqrt {\sum_{i = 1}^{N} \left( {x_{i} - \overline{x}} \right)^{2} } \sqrt {\sum_{i = 1}^{N} \left( {y_{i} - \overline{y}} \right)^{2} } }}$$

**Table 6:** Comparison of evaluation indicators before and after denosing.

	MSE	Correlation coefficient
Before denoising	0	0.465
After denoising	0	0.477

Utilizing wavelet denoising techniques for the processing of the two sets of monitoring data, a comparative analysis of the denoised data reveals prominent positions of signal variation. This enables the identification of locations where uneven settlement occurs.

**Figure 8:**
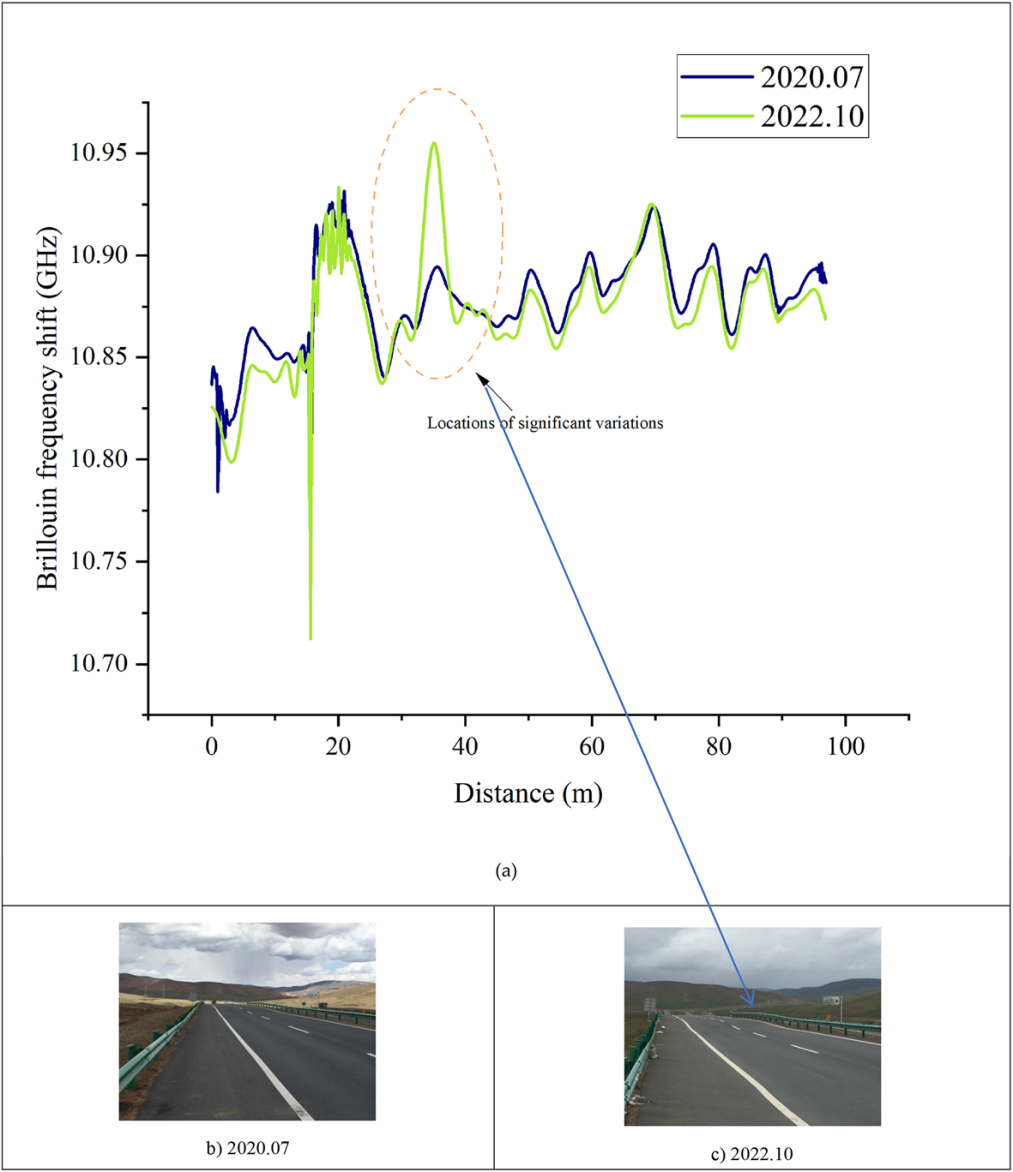
Comparison of the two monitoring datasets.

As illustrated in the Fig. 8, through the comparison of the fiber optic monitoring data for the same section in 2020 and 2022, both sets of data have undergone wavelet denoising. It can be observed that significant changes occur at the position of 38 m. Through on-site comparison, it was found that the field embankment at this location exhibited uneven settlement.

## Conclusions

This study tackles the unique challenges posed by fiber optic monitoring data of roadbed settlement in high-altitude and cold regions. Through the application of wavelet denoising theory and numerical simulations, the following conclusions are drawn:

The efficacy of wavelet denoising techniques in processing fiber optic monitoring data of roadbed settlement in cold regions is unequivocally demonstrated.

In light of the background of fiber optic monitoring data in high-altitude and cold regions, a multi-index fusion parameter, OPT, is proposed to address the issue of extremely low RMSE values in the monitoring signals. By observing the rate of change of the OPT parameter, a comprehensive consideration of the convergence and divergence of multiple indicators is achieved to determine the optimal decomposition level. However, it is noteworthy that while the OPT index provides valuable insights, further research is needed to ascertain its applicability and reliability under varying environmental conditions. Additionally, for non-permafrost regions where RMSE indicators exhibit significant fluctuations, alternative methods for selecting the optimal decomposition level may be more suitable.

The research findings indicate that in scenarios where the type of environmental noise is unknown and establishing a rational noise model poses challenges, fixed threshold methods offer certain advantages over other denoising thresholds. Nonetheless, it is imperative to acknowledge the potential drawbacks of this approach, namely, the variability of environmental noise over time or across different conditions, which may render the denoising effects unstable. Future research efforts should focus on the establishment of environmental noise models in permafrost regions.

The selection of Daubechies wavelets as the mother wavelet for processing fiber optic monitoring data in cold regions is based on their superior local frequency analysis capabilities and their ability to preserve important signal features during denoising, along with their widespread use in engineering applications. However, it is noted that the wavelet basis function database utilized in this study is established based on existing engineering precedents, and engineering case studies specific to permafrost regions are relatively scarce. Therefore, engineering expertise is indispensable in selecting wavelet functions, and as engineering practices in permafrost regions continue to evolve, more suitable wavelet basis functions may be identified.

## Data Availability

The datasets used and/or analysed during the current study available from the corresponding author on reasonable request.
